# Characterization of Small Interfering RNAs Derived from *Sugarcane Mosaic Virus* in Infected Maize Plants by Deep Sequencing

**DOI:** 10.1371/journal.pone.0097013

**Published:** 2014-05-12

**Authors:** Zihao Xia, Jun Peng, Yongqiang Li, Ling Chen, Shuai Li, Tao Zhou, Zaifeng Fan

**Affiliations:** 1 State Key Laboratory of Agro-biotechnology and Ministry of Agriculture Key Laboratory for Plant Pathology, China Agricultural University, Beijing, China; 2 Ministry of Agriculture Key Laboratory of Integrated Pest Management on Tropical Crops, Environmental and Plant Protection Institute, Chinese Academy of Tropical Agricultural Sciences, Haikou, Hainan, China; Department of Primary Industries and Fisheries, Australia

## Abstract

RNA silencing is a conserved surveillance mechanism against viruses in plants. It is mediated by Dicer-like (DCL) proteins producing small interfering RNAs (siRNAs), which guide specific Argonaute (AGO)-containing complexes to inactivate viral genomes and may promote the silencing of host mRNAs. In this study, we obtained the profile of virus-derived siRNAs (vsiRNAs) from *Sugarcane mosaic virus* (SCMV) in infected maize (*Zea mays* L.) plants by deep sequencing. Our data showed that vsiRNAs which derived almost equally from sense and antisense SCMV RNA strands accumulated preferentially as 21- and 22-nucleotide (nt) species and had an adenosine bias at the 5′-terminus. The single-nucleotide resolution maps revealed that vsiRNAs were almost continuously but heterogeneously distributed throughout the SCMV genome and the hotspots of sense and antisense strands were mainly distributed in the HC-Pro coding region. Moreover, dozens of host transcripts targeted by vsiRNAs were predicted, several of which encode putative proteins involved in ribosome biogenesis and in biotic and abiotic stresses. We also found that *ZmDCL2* mRNAs were up-regulated in SCMV-infected maize plants, which may be the cause of abundant 22-nt vsiRNAs production. However, *ZmDCL4* mRNAs were down-regulated slightly regardless of the most abundant 21-nt vsiRNAs. Our results also showed that SCMV infection induced the accumulation of *AGO2* mRNAs, which may indicate a role for AGO2 in antiviral defense. To our knowledge, this is the first report on vsiRNAs in maize plants.

## Introduction

RNA silencing is a conserved antiviral defense mechanism in plants. The antiviral silencing can be triggered by viral double-stranded RNA (dsRNA) and highly structured single-stranded RNA (ssRNA), which can be recognized and cleaved by Dicer-like (DCL) proteins and processed into virus-derived small interfering RNAs (vsiRNAs) that vary in length from 21 to 24 nucleotides (nt) in virus-infected plants [1]–[4]. The visRNAs are then loaded into Argonaute (AGO)-containing complexes known as RNA-induced silencing complexes (RISCs), promoting the degradation of both genomic and subgenomic viral RNAs and the silencing of host mRNAs in a sequence-specific manner [5]–[9]. Two classes of vsiRNAs are generated during virus infections: primary siRNAs, which derived from DCL-mediated cleavage of an initial trigger RNA, and secondary siRNAs, whose biogenesis requires an RNA dependent RNA polymerase (RDR) [10]–[13].

DCL4 and DCL2 play key roles in the generation of vsiRNAs derived from positive-strand RNA viruses to produce 21- and 22-nt vsiRNAs, respectively [11], [12], [14]. Plants infected with positive-strand RNA viruses mainly accumulate 21-nt vsiRNAs processed by DCL4, but when the activity of DCL4 is reduced or inhibited by viruses, DCL2, as the substitute, is known to produce 22-nt vsiRNAs [12], [14]–[17]. However, recent findings have suggested that there is a difference between 21- and 22-nt vsiRNAs in antiviral defense, and DCL2-dependent 22-nt vsiRNAs alone do not guide efficient silencing [18]. In addition, it is demonstrated that the production of viral secondary siRNAs mainly depends on host RDR1, RDR2, or RDR6 in *Arabidopsis* infected by distinct positive-strand RNA viruses [12], [13], [19]–[21]. Moreover, it was reported that RDR1 and RDR6 exhibited specificity in targeting the genome sequences of *Cucumber mosaic virus* (CMV) in amplifying viral secondary siRNAs [13]. vsiRNAs are associated with specific AGO complexes to function in RNA silencing [22]–[25]. In plants, the recruiting small RNAs of a particular AGO complex is preferentially, but not exclusively, dictated by their 5′-terminal nucleotides [18], [25]–[27]. In *Arabidopsis*, there are higher levels of viral RNA accumulation in hypomorphic *ago1*, null *ago2* and *ago7* mutants, and AGO1, AGO2, and AGO5 proteins can bind vsiRNAs, suggesting an antiviral role for these AGOs [17], [18], [28]–[33]. Moreover, it was reported that vsiRNAs could be recruited into AGOs 1, 2, 3, 5, 7 and 10, which were demonstrated to exhibit *in vitro* slicer activity [25]. Recent studies also revealed that AGO2 plays an antiviral role in *Nicotiana benthamiana* [34]. Other components involved in RNA silencing also participate in antiviral defense in plants, including dsRNA-binding protein 4 (DRB4), suppressor of gene silencing 3 (SGS3) and HUA ENHANCER 1 (HEN1) [11], [22], [35]–[39].

It was predicted previously that vsiRNAs could target host transcripts at post-transcriptional level, as endogenous miRNAs or siRNAs. To date, only a few studies have provided experimental evidence to verify the targeting of host genes, although many host transcripts potentially targeted by vsiRNAs have been predicted using bioinformatics [6], [7], [40]. Early studies suggested that some of the vsiRNAs may target host transcripts for post-transcriptional regulation by BLAST search and 5′ RACE [3], [41], [42]. Recently, two research groups confirmed that vsiRNA derived from the Y-satellite of CMV could specifically and directly cleave *ChlI* mRNA in *N. benthamiana* and modulate the virus disease symptoms [6], [7]. Moreover, it was demonstrated that siRNA containing the pathogenic determinant of a chloroplast-replicating viroid guided the degradation of the mRNA encoding the chloroplastic heat-shock protein 90 as predicted by RNA silencing [43]. It was also reported that vsiRNAs promoted the silencing of host mRNAs in a sequence-specific manner by degradome analysis and 5′ RACE [9].

*Sugarcane mosaic virus* (SCMV), a member of the genus *Potyvirus*, can infect various crops (e.g., sugarcane, sorghum, and maize) which leads to symptoms such as mosaic, chlorosis and dwarfing, and causes considerable losses in different field crops in the world [44], [45]. Our previous studies showed that SCMV was the major causal agent of maize dwarf mosaic disease in China, and the Beijing isolate (SCMV-BJ) belonged to the prevalent strain [46]. It was reported that SCMV infection could elicit the accumulation of *RDR1* mRNA, and silenced *RDR1* maize plants were more susceptible to SCMV infection [47]. Co-expression assay demonstrated that the HC-Pro encoded by SCMV suppressed the RNA silencing induced by sense RNA and dsRNA, and down-regulated the accumulation of *RDR6* mRNA [48]. These results suggested that RDR1 and RDR6 may be involved in SCMV infection and plant antiviral defense. Other reports have investigated the interaction between SCMV and maize, including protein-protein interaction and the possible genes involved in the defense responses to SCMV infection [49]–[54]. However, the roles of the vsiRNAs played in the interaction between SCMV and maize were still unknown. In this study, the profile of vsiRNAs derived from SCMV in infected maize (*Zea mays* L.) plants was obtained by deep sequencing. We analyzed the characters of vsiRNAs and predicted the targets of some vsiRNAs. Moreover, the relative accumulation level of *ZmDCLs* and *ZmAGO2* mRNAs in SCMV-infected maize plants were detected.

## Results

### 21- and 22-nt vsiRNAs accumulated at high levels in maize plants inoculated with SCMV

**Figure 1:**
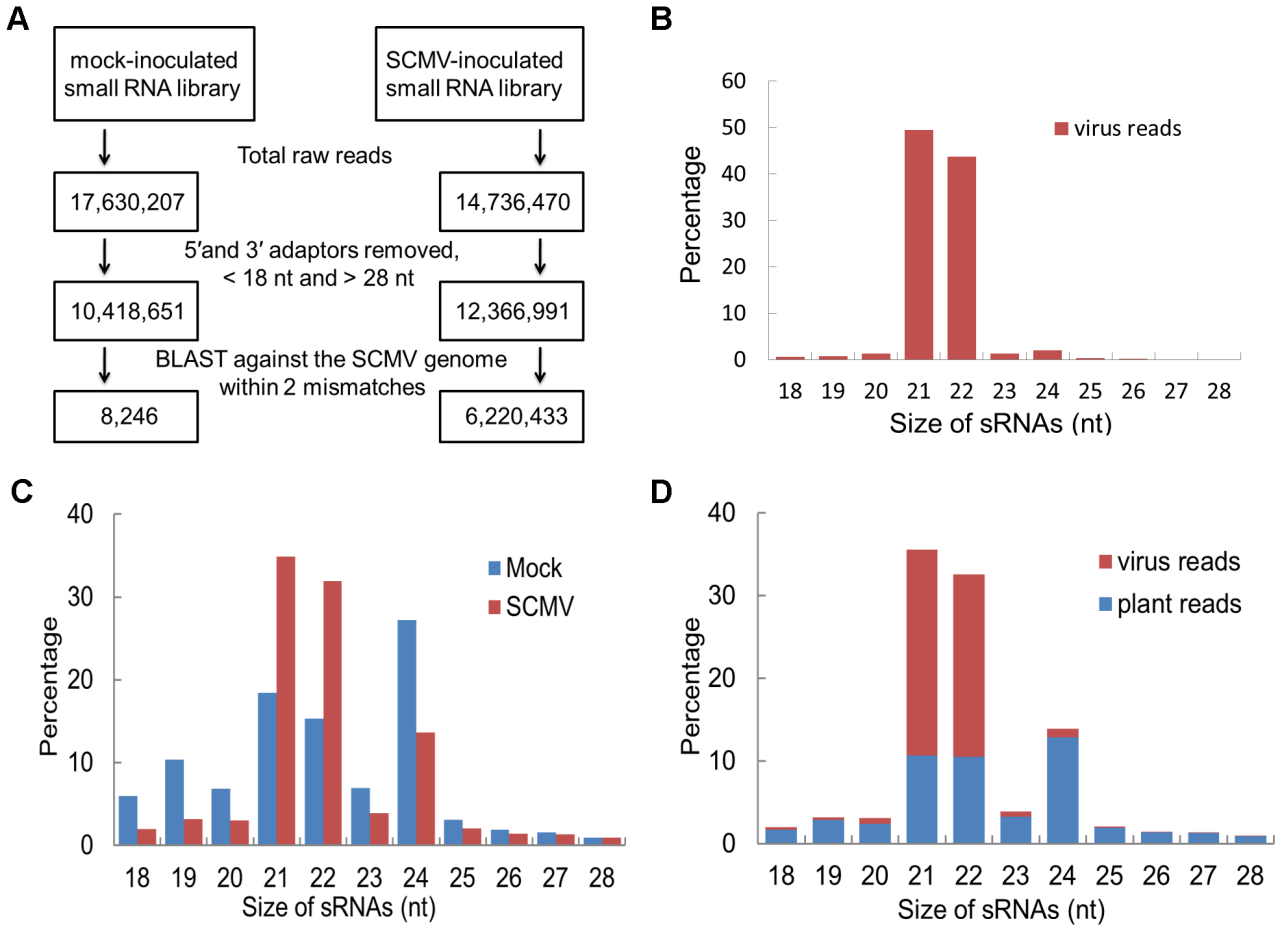
21- and 22-nt vsiRNAs accumulated at high levels in SCMV-inoculated maize. A: Diagram showing the stepwise computational extraction of vsiRNA reads from small RNA libraries recovered from mock-inoculated and SCMV-inoculated systemic leaves. B: Histogram representation of total vsiRNA reads in each size class. C: Size distribution of total small RNAs in libraries prepared from either mock-inoculated or SCMV-inoculated maize plants. D: Size distribution of total small RNAs in the library from SCMV-inoculated maize plants.

The profile of vsiRNAs can help to decipher the mechanisms and components involved in their biogenesis and function. To obtain the profile of vsiRNAs produced during SCMV infection, small RNAs obtained from maize plants inoculated with SCMV or with phosphate buffer (mock) were analyzed by deep sequencing using the Illumina Solexa platform. A total of 17,630,207 and 14,736,470 reads were obtained from small RNA library of either mock- or SCMV-inoculated maize plants, respectively (Figure 1A). Reads ranging from 18- to 28-nt were mapped to the viral genome in sense and antisense orientations. The sequences within 2 mismatches were regarded as vsiRNAs in the libraries (Figure 1A). In total, 6,220,433 vsiRNA reads were identified in SCMV-inoculated maize plants, accounting for more than half of 18–28 nt reads. However, only 8,246 reads matched to the SCMV genome in the mock-inoculated library, which corresponded to approximately 0.08% of 18–28 nt reads (Figure 1A). In SCMV-infected maize plants, 21- and 22-nt vsiRNAs accumulated to high levels, representing 49.42% and 43.79% of total vsiRNAs, respectively (Figure 1B), which suggested that the maize homologue of DCL4 and DCL2 may be the predominant Dicer ribonucleases involved in vsiRNA biogenesis. We then compared the overall profile of small RNAs between mock- and SCMV-inoculated libraries. The results showed that 21- and 22-nt reads increased significantly in the SCMV-inoculated library, while 24-nt reads decreased (Figure 1C). Interestingly, the increase of the 21- and 22-nt small RNAs was mainly attributed to the accumulation of vsiRNAs (Figure 1D), suggesting that SCMV infection produced amounts of vsiRNAs and the high levels of vsiRNAs seemed to be a result of the antiviral RNA silencing mechanism or a specific SCMV-host interaction.

### The characteristics of vsiRNAs

**Figure 2:**
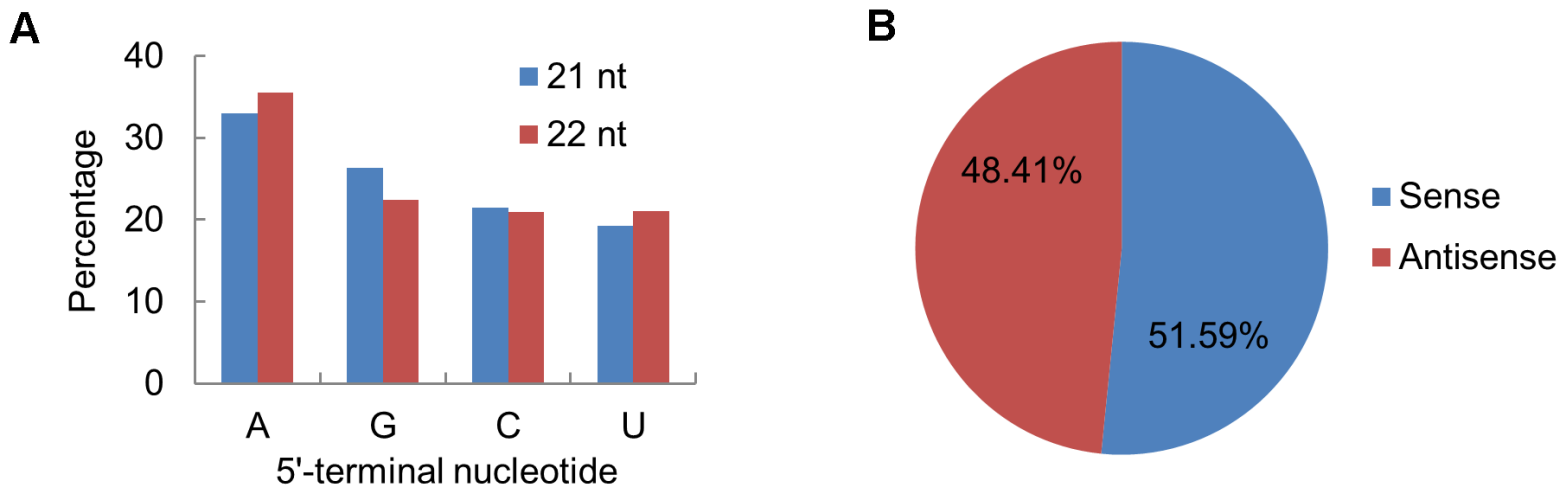
Relative frequency of 5′-terminal nucleotide of vsiRNAs and accumulation of sense and antisense vsiRNAs. A: Relative frequency of distinct 5′-terminal nucleotides in 21- and 22-nt vsiRNAs of SCMV-inoculated library. B: Accumulation of sense and antisense vsiRNAs. Percentage for each class of vsiRNAs from the SCMV-inoculated library is shown within the pie graph.

In *Arabidopsis*, it has been reported that the selective loading of small RNAs into specific AGOs is influenced by their 5′-terminal nucleotides [26]. To determine potential interactions between vsiRNAs with distinct AGO complexes, we analyzed the relative abundance of vsiRNAs according to their 5′-terminal nucleotides (Figure 2A). For the 21- and 22-nt vsiRNAs, A was the most abundant nucleotide at the 5′-end (32.99% and 35.50%, respectively), while U was the least abundant (19.25% and 21.07%, respectively). These results suggested that 21- and 22-nt vsiRNAs might be potentially loaded into diverse AGO-containing complexes with most of vsiRNAs preferentially loaded into AGO2 and/or AGO4, which showed a preference for A [26].

**Figure 3:**
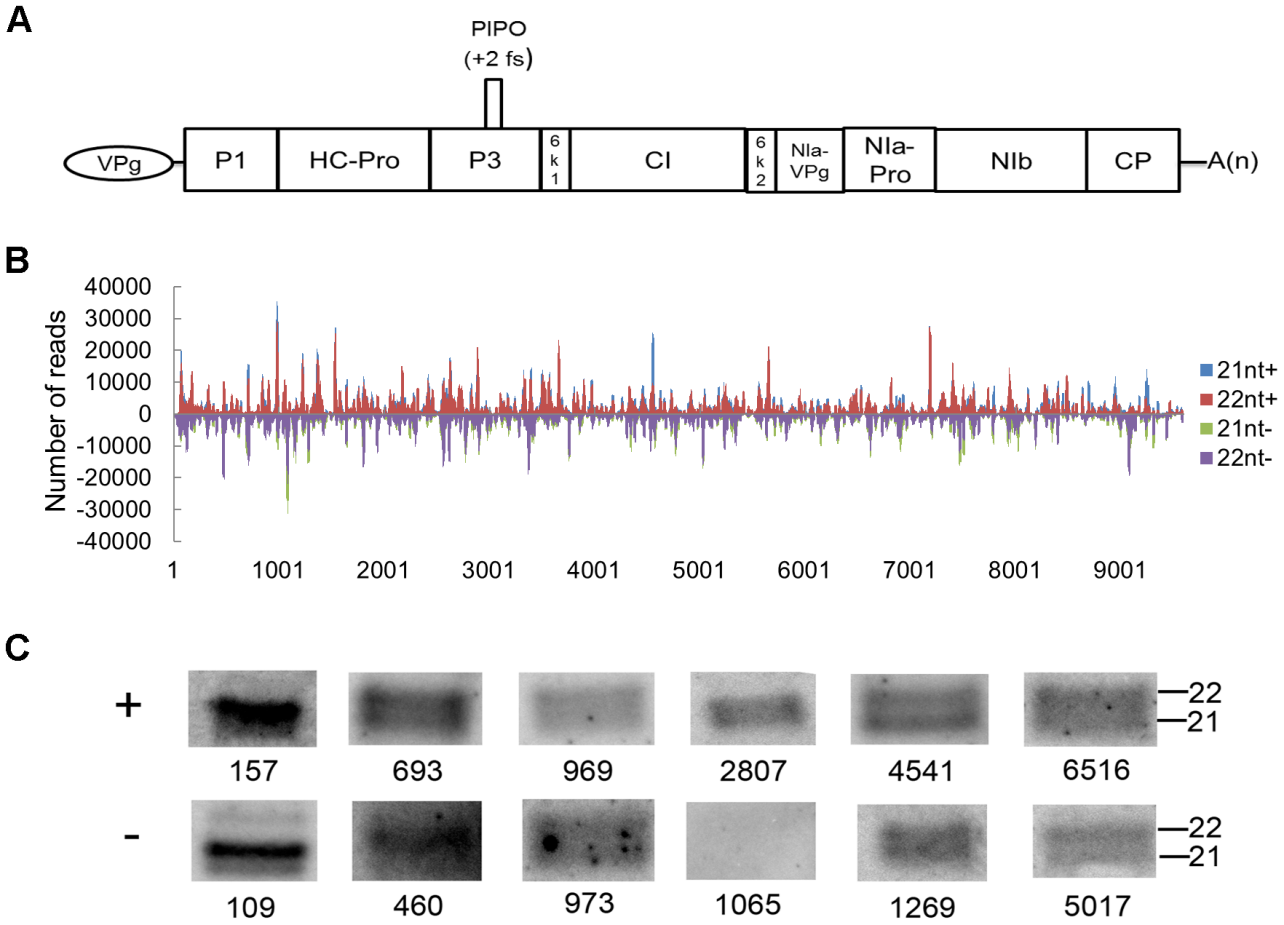
Profile of vsiRNAs derived from SCMV-inoculated library. A: Schematic diagram of SCMV genome. B: Maps of 21- and 22-nt vsiRNAs from SCMV-inoculated maize plants at single-nucleotide resolution. The graphs plot the number of 21- and 22-nt vsiRNA reads at each nucleotide position of the 9595-nt SCMV genome; Bars above the axis represent sense reads starting at each respective position; those below represent antisense reads ending at the respective position. C: Northern blotting of vsiRNAs from distinct regions. “+” indicates vsiRNAs derived from sense strand of SCMV genome; “-” indicates vsiRNAs derived from antisense strand of SCMV genome.

To explore the origin of the vsiRNAs, the polarity distribution of vsiRNAs was further characterized. Almost equivalent amounts of sense (51.59%) and antisense (48.41%) vsiRNAs suggested that vsiRNAs derived from both sense and antisense SCMV RNA strands to a similar extent (Figure 2B). To examine the genomic distribution of the vsiRNAs, 21- and 22-nt vsiRNA sequences were mapped along the SCMV genome (Figure 3A and 3B). The single-nucleotide resolution maps indicated that vsiRNAs from both polarities were almost continuously but heterogeneously distributed throughout the SCMV genome (Figure 3B and Table S1). To better understand the hotspots of vsiRNAs distribution, we counted and summed up the reads of single-nucleotide resolution maps of 21–24 nt vsiRNAs, and defined the region that the number of at least 21 consecutive single-nucleotide reads should be not less than 30,000 as a hotspot (Table S2). Further estimation of the vsiRNA-generating hotspots showed that the number of hotspots derived from the sense strand was more than that from antisense strand, and the region corresponding to HC-Pro contained more hotspots (Figure 3B and Table S2). Moreover, we calculated the GC content of each hotspot on both sense and antisense strand and found that the GC content of most hotspots were less than 50% (Table S2), not as high (GC content within hotspots) as reported [55], [56]. The results we obtained also indicated that most prominent peaks of sequence abundance corresponding to 21-nt vsiRNAs usually localized to the same genomic regions as peaks corresponding to 22-nt vsiRNAs (Table S1). Nevertheless, the positions 4540-4561 on sense strand and positions 460-481 on antisense stand had a preference to 21-nt and 22-nt, respectively (Table S2). The results indicated that different DCLs have a similar but slightly different targeting preference toward the same regions along the viral genome.

There were large amounts of vsiRNAs accumulated in the host plants when virus infection triggered the RNA silencing mechanism. To confirm the existence of vsiRNAs, approximately 15 µg of total RNAs was used to analyze the accumulation of vsiRNAs derived from different SCMV genome positions by Northern blotting. The results showed that there were almost equivalent 21- and 22-nt vsiRNAs in the SCMV-infected maize plants, except that vsiR157 (+), vsiR109 (-) and vsiR460 (-) had a preference for 22-nt and vsiR4541 (+) had a preference for 21-nt (Figure 3C), which was consistent with the results of deep sequencing (Table S1). These results indicated that there were indeed large amounts of vsiRNAs accumulation in SCMV-infected maize plants and DCLs played different roles in processing different positions of viral RNAs. However, vsiR1065 (-) hardly had any detectable signal, which implied that little such vsiRNAs accumulated.

### Plant transcripts targeted by vsiRNAs

MiRnada is an algorithm for finding genomic targets for miRNAs [57]. In this study, we used this method to identify maize mRNAs targeted by vsiRNAs derived from SCMV. Due to the vast variety of vsiRNAs, only some vsiRNAs with high abundant reads were selected (Table S3) and only the targets whose scores were not less than 180 were presented in Table S4. The results showed that most vsiRNAs derived from the sense strand had only one target in the given condition, while most vsiRNAs derived from antisense strand had more than one targets (Table S4), indicating that the vsiRNAs from different strands might play distinct roles in regulating the expression of host transcripts. Moreover, some vsiRNAs had multiple targets, for example, vsiR2304 (+), vsiR4318 (+), vsiR8469 (+), vsiR699 (-) and vsiR7454 (-), and in most cases, they could target different transcripts from one gene (Table S4), which suggested that they might have versatile functions in different physiological pathways. The results also showed that predicted targets were involved in many different physiological pathways, including gene expression, energy metabolism, signal transduction, transcriptional regulation and cell defense (Table S4). The broad range of targets suggested that the identified vsiRNAs possibly played significant roles in SCMV-inoculated maize plants.

**Figure 4:**
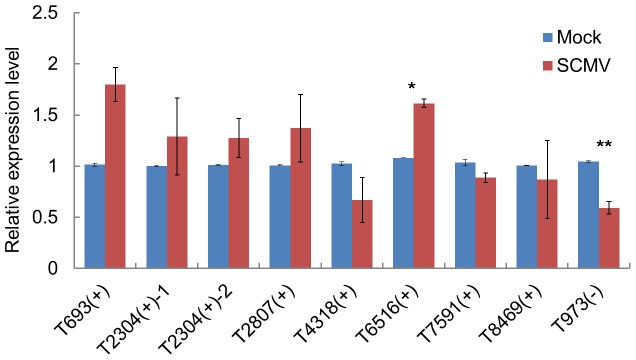
The expression level of the predicted target mRNAs of vsiRNAs in mock- (blue) and SCMV-inoculated (red) maize plants. For each target, the asterisk(s) indicates significant differences (*P<0.05; **P<0.01) of SCMV-inoculated versus mock-inoculated maize plants. The information and primer sequences of the predicted targets were listed in Table S5.

SiRNAs are known to down-regulate targets at the post-transcriptional level. To determine whether vsiRNAs from SCMV promoted the degradation of target transcripts, quantitative real-time reverse transcription-polymerase chain reaction (qRT-PCR) was carried out to examine the accumulation of target transcripts. Some predicted targets (whose corresponding vsiRNAs had higher number reads) that had high scores, were selected to perform qRT-PCR, except for T2807 (+) and T973 (-), whose score were 179 (Table S5). The accumulation of T973 (-) was significantly down-regulated in SCMV-infected maize plants, while T6516 (+) was up-regulated and there was no significant change in other predicted targets (Figure 4). The results indicated that these targets might be involved in several pathways rather than only be regulated by vsiRNAs at the post-transcriptional level.

**Figure 5:**
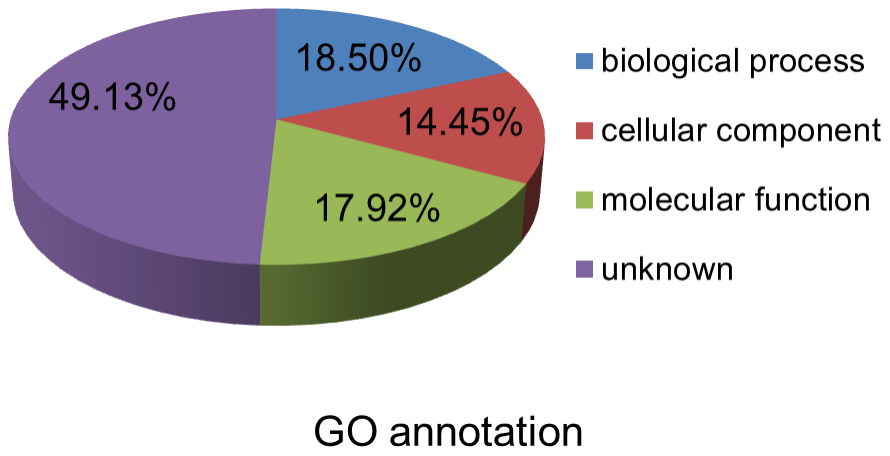
Functional classification of some predicted vsiRNAs target transcripts according to BLAST2GO. The GO classification includes biological process, molecular function and cellular component. The detailed GO annotation information of each target transcript was listed in Table S4.

To understand the roles of the predicted vsiRNA target genes in maize, the target gene sequences were used to query the Gene Ontology (GO) database [58]. Since the scores of the majority of the predicted targets were low, targets with scores not less than 180 were analyzed with GO annotations (Table S4). The vsiRNA target genes were grouped into three root GO categories: molecular function (MF), biological process (BP) and cellular components (CC) (Figure 5). In addition to unknown genes (accounted for 49.13% vsiRNA target genes which showed no matches in the GO database), the most abundant target genes were classified as BP GO term (32/173), including reproduction, cellular process and metabolic process functions, followed by MF GO term (31/173), which consisted of binding and catalytic activity function (Figure 5 and Table S4). Other targets were classified as CC GO term (25/173) (Figure 5), and the secondary classification of these targets were overlapped (Table S4), suggesting that they may play different roles as cellular components.

### Differential expression of *ZmDCLs* and *ZmAGO2* mRNAs after SCMV infection

Our results demonstrated that there was abundant 21- and 22-nt vsiRNAs accumulation after SCMV infection. To gain insights into the effects of SCMV infection on the RNA silencing pathways, we characterized the accumulation of *ZmDCLs* mRNAs involved in the biogenesis of vsiRNAs using qRT-PCR. The results indicated that the accumulation of *ZmDCL2* mRNA was significantly up-regulated, while *ZmDCL4* was down-regulated and there were no significant differences in the levels of *ZmDCL1*, *ZmDCL3a* and *ZmDCL3b* mRNAs between mock- and SCMV-inoculated maize plants (Figure 4A). Considering the 5′-terminal nucleotides of most of vsiRNAs were A, we explored the expression of *ZmAGO2* mRNA. The results showed that the accumulation of *ZmAGO2* mRNA was significantly induced after SCMV infection (Figure 4B), indicating a role for ZmAGO2 in antiviral defense. Taken together, these results might represent a distinct mechanism involved in the interaction between SCMV and maize plants.

## Discussion

RNA silencing is a small RNA-mediated repression mechanism of gene regulation in eukaryotes and plays a critical role in the defense against viruses in plants. Virus infection triggers the production of vsiRNAs in infected plant cells. In this study, a Solexa-based deep-sequencing approach was used to profile vsiRNAs populations from SCMV-inoculated maize plants.

**Figure 6:**
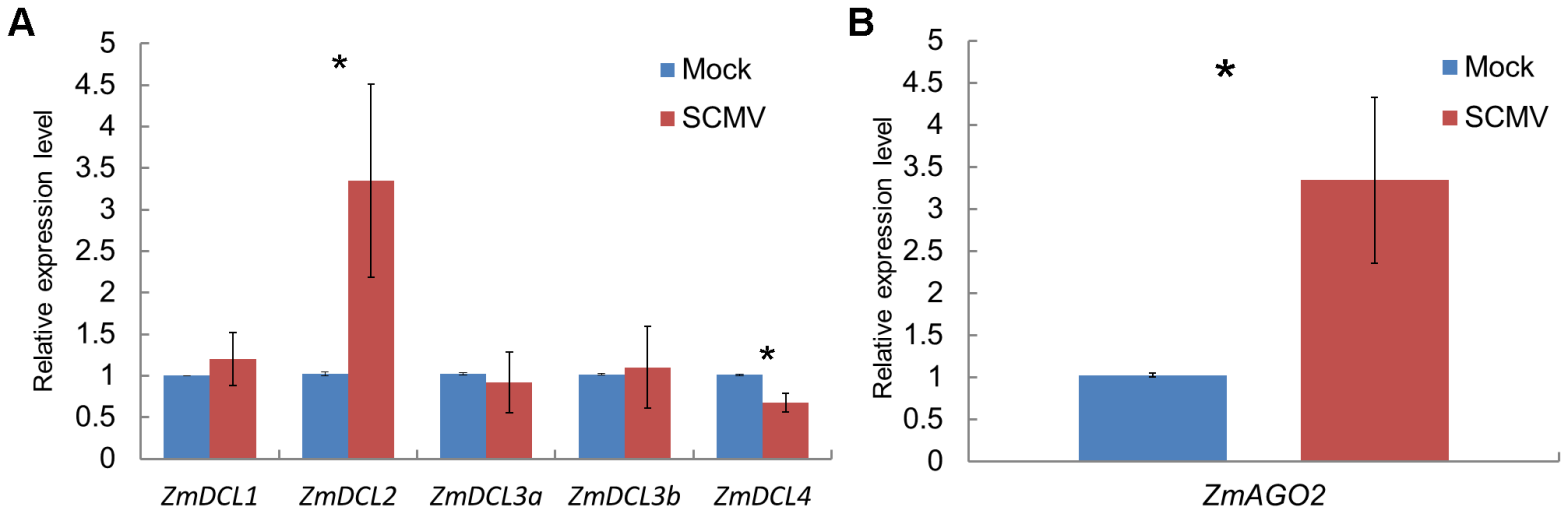
qRT-PCR analysis of the expression of maize *DCLs* and *AGO2* mRNAs in mock- (blue) and SCMV-inoculated (red) maize plants. For each gene, asterisk indicates significant differences (*P<0.05) of SCMV-inoculated versus mock-inoculated maize plants. The information and primer sequences used for amplification of *ZmDCLs* and *ZmAGO2* were listed in Table S6.

Sequence analysis of the deep-sequencing data revealed that SCMV infection triggered the production of large amounts of vsiRNAs, which accounted for 50.30% of the 18–28 nt reads. Our results also showed that there were more abundant small RNAs accumulation in SCMV-inoculated maize plants than that in mock-inoculated plants by ethidium bromide (EtBr) staining of size-separated RNAs (data not shown). In positive-strand RNA virus-infected plants, DCL4-dependent 21-nt vsiRNAs are the most abundant species [12], [14], [16,], whereas DCL2-dependent 22-nt vsiRNAs accumulated to higher levels in the absence of DCL4 [15]. However, 22-nt vsiRNAs accumulated predominately in *Tobacco rattle virus* (TRV)-infected *N. benthamiana* plants and *Cotton leafroll dwarf virus* (CLRDV)-infected cotton plants [10], [59]. In TRV-infected *N. benthamiana* leaves, TRV-derived siRNAs of 22-nt (44.7%) were cloned to the same extent as 21-nt (42.5%), whereas 21-nt siRNA species were overrepresented (65.2%) in TRV-infected *Arabidopsis* [10]. Different size class distribution of vsiRNAs suggested the difference of the biosynthetic pathways of siRNAs in *N. benthamiana* and those in *Arabidopsis* [10]. In our study, 21- and 22-nt vsiRNAs accumulated at high levels (49.42% and 43.79%, respectively) in SCMV-infected maize plants, suggesting that DCL4 and DCL2 worked redundantly and, perhaps, synergistically in the production of vsiRNAs, which is consistent with the model that cooperative interaction between DCL4 and DCL2 was necessary during systemic antiviral silencing in TuMV-infected *Arabidopsis* [12], as all the experimental samples we used were maize systemic leaves. In SCMV-infected maize plants, *ZmDCL2* mRNA was up-regulated (Figure 6A), confirming the role ZmDCL2 played in the production of vsiRNAs. Nevertheless, the *GhDCL2* mRNA was down-regulated in CLRDV-infected cotton plants and the predominance of 22-nt vsiRNAs associated with CLRDV infection would be hypothesized to be the result of GhDCL2 activity [59]. Surprisingly, though *ZmDCL4* mRNA was down-regulated (Figure 6A), yet there existed the most abundant 21-nt vsiRNAs accumulation in SCMV-infected maize plants (Figure 1B), indicating that ZmDCL4 still played the major role in biosynthesis of vsiRNAs. In previous reports, TCV infection was associated with an abundance of 22-nt vsiRNAs, which seemed to be related to the activity of the suppressor protein P38 that could indirectly block AtDCL4 activity by suppressing AGO1 function [15], [28]. Although HC-Pro had been proved to function as a viral suppressor of RNA silencing (VSR) and down-regulate the accumulation of 3′ secondary siRNA and *RDR6* mRNA [48], the possible correlation between HC-Pro and DCLs is still unknown.

Interestingly, as demonstrated by deep sequencing (Table S1) and Northern blotting results (Figure 3C), hotspots of each vsiRNA size class typically co-localized within the same regions of SCMV genome in SCMV-inoculated maize plants, especially 21- and 22-nt vsiRNAs, indicating similar, although hierarchical, targeting affinities among the DCL enzymes [37]. It has been reported that DCL activities could be favored by a higher GC content within hotspots rendering dsRNA structures more stable [20], [55], [56]. In this study, the GC content of each hotspot of sense and antisense strand had been obtained (Table S2), while there were no obvious correlations between higher GC content and hotspots of vsiRNAs. To date, it is not yet clear what structural features ultimately influence the accessibility, affinity or processing of DCLs [32], [37].

In previous reports, AGO1 played a dominant role in defending against RNA viruses [31]–[33]. However, in SCMV-infected maize plants, the vsiRNAs with a 5′-terminal U, which would be loaded into AGO1, accounted for the smallest proportion (Figure 2A), suggesting that this may be a new mechanism of weakening RNA silencing against SCMV. Recently, more and more reports showed that AGO2 plays an antiviral role in different plant spices [29], [30], [34]. Moreover, the vsiRNAs loaded into different AGOs to form RISC had been demonstrated by AGO immunoprecipitates test [18], [25]. In this study, the majority of the 21- and 22-nt vsiRNAs in SCMV-inoculated library showed a bias for sequences beginning with a 5′-terminal A, indicative of their association with AGO2 and/or AGO4. Interestingly, our data showed that SCMV infection induced the accumulation of *ZmAGO2* mRNA (Figure 6B), which further increases the possibility that ZmAGO2 participated in antiviral defense. In addition, the presence of large amounts of vsiRNAs whose 5′-terminal nucleotides were G or C accumulated, revealing that other AGOs may also be recruited to form different RISCs and involved in antiviral defense.

Polarity distribution analysis of the sequenced vsiRNAs demonstrated the presence of approximately equal ratios of sense and antisense vsiRNAs (Figure 2B), indicating that most of vsiRNAs would be produced from dsRNA precursors comprised of sense and antisense strands. However, this could not explain the existence of hotspots and non-hotspots, because each position on the viral genome was a potential cleavage site in producing vsiRNA [40]. Moreover, the hotspots of sense and antisense strand were clustered in different regions of SCMV genome (Figure 3B). Although it had been suggested that dsRNA-like secondary structures within the single-stranded viral RNA were more likely to be the main source of vsiRNAs than dsRNA replication intermediates [4], [12], [13], [41], [60], it was not successful to find significant correlations between hotspots and regions predicted to adopt a potential hairpin structure in this study (data not shown). Recently, it was reported that (-) RNA was not accessible to antiviral RNA silencing, which could be another explanation for plants infected with different RNA viruses, e.g. the TBSV-related CymRSV, revealed a strong bias for the generation of vsiRNAs from the (+) RNA [25]. However, it is not clear whether this mechanism also functions in SCMV-infected maize plants. Our results suggested that most vsiRNAs of non-hotspots might be produced and subsequently degraded by unknown mechanisms, which need to be further investigated.

It is unclear if all the vsiRNAs produced in the host cell can be incorporated into AGO-containing RISCs, and it remains to be established whether vsiRNAs can be recruited into all the AGO family members [40]. Recently, the findings from a research seemed to give us an answer that the majority of the vsiRNAs derived from TBSV were inefficient in guiding the formed RISC and specific vsiRNAs could be recruited into AGOs 1, 2, 3, 5, 7 and 10 of *Arabidopsis*, which were demonstrated to exhibit *in vitro* slicer activity [25]. In the presence of vsiRNAs, only a distinct number rather than a broad variety of cleavage products were obtained, revealing that only some distinct vsiRNAs may be highly effective [25]. In another report, only a few cleavage sites were found in the viral genomes by degradome analysis, and vsiRNA hotspots were not directly associated with cleavage sites [9]. It has been reported that vsiRNAs generated from hotspots, in spite of their much greater abundance, do not exhibit a greater efficiency than those from non-hotspots regions [40], [60]. Thus, we speculated that only some distinct vsiRNAs would be incorporated into specific AGO-containing RISC and involved in the antiviral silencing. Some cleavage sites on the SCMV genome directed by vsiRNAs have been found (data not shown), but the functional vsiRNAs of antiviral response remains a subject of further investigation.

Previous studies suggested that vsiRNAs can target host mRNAs at post-transcriptional level [6], [7], [9], [43]. In this study, most of the predicted targets were not down-regulated (Figure 4), inferring that many factors, for example, virus-encoded silencing suppressors and abundance of vsiRNAs, might affect the functionality of vsiRNAs and hence restrict their regulatory potential on host targets *in vivo* [40]. In addition, vsiRNAs might regulate host targets by translation inhibition, not only cleavage of mRNAs, similar to the characteristics of miRNAs [61]-[63]. Moreover, the possibility cannot be excluded that SCMV infection could induce over-expression of some transcripts in a non-RNA silencing-related pathway [9].

## Materials and Methods

### Ethics statement

No specific permission is required for these sampling locations in this study, and do not need to provide details on why this is the case. Also, we did not require ethical approval to conduct this study as we did not handle or collect any animal species considered in any animal welfare regulations and no endangered or protected species were involved in the samplings or the experiments.

### Plant growth, virus source and small RNA sequencing

Maize (*Zea mays* L.) inbred line Zong 31 plants were grown in growth chambers (28 °C day and 22 °C night, 16 h light and 8 h dark cycles) for plant growth and virus inoculation. SCMV-BJ (accession number AY042184) were isolated from diseased maize in the northern suburbs of Beijing [46] and maintained at -80 °C. At 8 days post-inoculation (dpi), when the newly developed leaves started to show viral symptoms, the systemically infected leaves were harvested (16 days after maize germination). With each treatment, the systemic leaves of at least 15 maize seedlings were pooled for RNA extraction. Total RNA was extracted using Trizol reagent (Invitrogen, Carlsbad, CA, USA) for qRT-PCR, small RNA sequencing and Northern blotting. For deep sequencing, total RNA concentration was examined with a spectrophotometer (Nanodrop ND-2000, ThermoFisher Scientific, Wilmington, DE, USA), and RNA sample integrity was verified by a Bio-Analyzer 2100 (Agilent Technologies, Waldbronn, Germany). Then, in brief, total RNA was separated through 17% denaturing polyacrylamide gels and small RNAs of 15–36 nt were recovered. After that, RNA adaptors were ligated to these small RNAs followed by reverse transcription into cDNAs. These cDNAs were finally amplified by PCR and subjected to Solexa/Illumina sequencing by SBC (Shanghai Biotechnology Corporation, Shanghai, China).

### Bioinformatic analyses of small RNA sequences

Small RNA sequences were computationally analysed by a set of Perl scripts from datasets generated from Illumina sequencing data. The adapter sequences were trimmed from raw reads and small RNAs between 18–28 nt in length were extracted. Only small RNA reads of sequences identical or complementary to SCMV genomic sequences within 2 mismatches were recognized as vsiRNAs (Figure 1A).

### Target Gene Prediction and Analysis

In this study, we adopted MiRnada to predict maize mRNAs targeted by vsiRNAs derived from SCMV [57]. Briefly, the criteria used were as follows: 1) No more than four mismatches between vsiRNA and target (G-U bases count as 0.5 mismatches), 2) No more than two adjacent mismatches in the vsiRNA/target duplex, 3) No adjacent mismatches in positions 2–12 of the vsiRNA/target duplex (5′-terminus of vsiRNA), 4) No mismatches in positions 10-11 of vsiRNA/target duplex, 5) No more than 2.5 mismatches in positions 1–12 of the vsiRNA/target duplex (5′-terminus of vsiRNA), 5) The predicted complementary structure between vsiRNA and target has a high minimal folding free energy (MFE) that must be no fewer than 75% of the best complementary structure.

The predicted target genes were aligned using BLAST (http://blast.ncbi.nlm.nih.gov/) and were mapped and annotated by BLAST2GO (version 2.5.0) [58]. The genes were characterized using GO terms, i.e., molecular function, biological process and cellular component.

### Northern blot analysis of vsiRNAs

Approximately 15 µg of total RNA (prepared as described above) was individually separated in a 15% urea polyacrylamide gel, electrophoretically transferred to Hybond-NX membrane (GE Healthcare, Buckinghamshire, UK) using a semi-dry transfer apparatus (Amersham Biosciences, Piscataway, NJ), and was chemically cross-linked via 1-ethyl-3-(3-dimethylaminopropyl) carbodiimide (EDC) [64]. For labeling reaction of probes, 1 µl of 10 µM probes, 2.5 µl of 10 x T4 PNK buffer (New England Biolabs), 3 µl of [γ-^32^P] ATP (∼10 µCi/µl), 17.5 µl of ddH_2_O and 1 µl of T4 Poly Nucleotide Kinase (New England Biolabs) were added (a total volume of 25 µl reaction) and kept in a water bath for 1 hour at 37°C. Probe sequences used for Northern blot analysis were shown in Table S7. Blots were pre-hybridized and hybridized at 42°C overnight using hybridization buffer (Sigma, USA). Post-hybridization washes were performed using 2 x SSC and 0.2% sodium dodecyl sulfate (SDS) at 42°C for 20 min for twice. Hybridization signals were detected by exposing blots to autoradiographic film.

### Quantitative Real-time RT-PCR

Total RNA was extracted from mock- and SCMV-inoculated maize leaves using TRIzol reagent (Invitrogen) and treated with 5 U of RNase-free DNAase I (TaKaRa Bio Inc., Dalian, China) at 37 °C for 30 min. The DNase I-treated total RNAs were recovered by ethanol precipitation. About 2 µg of total RNA was reverse-transcribed into cDNA and the qRT-PCR was performed as previously reported [49]. The sequence information of *ZmDCLs* and *ZmAGO2* refers to the report by Qian et al. [65]. The sequences of the primers used in the qRT-PCR experiments were listed in Table S5 and S6. The qRT-PCR experiments were performed to explore the expression of predicted targets; qRT-PCR amplification was also performed to determine the expression levels of *ZmDCL1, 2, 3a, 3b, 4* and *ZmAGO2*. The mean and standard errors were calculated over three biological and three technical replicates and the experimental data were subjected to *t*-test statistical analysis for these qRT-PCR experiments.

## Supporting Information

Table S1 Single-base resolution maps of 21–24 nt vsiRNAs originated from sense (+) and antisense (-) stand of SCMV genome in SCMV-inoculated maize plants.
(XLS)

Table S2 The characteristics of vsiRNA hotspots.
(XLS)

Table S3 The information of vsiRNAs selected for target prediction.
(XLS)

Table S4 Predicted maize mRNA targets of the selected vsiRNAs.
(XLS)

Table S5 The primers used for qRT-PCR amplification for predicted target transcripts.
(XLS)

Table S6 The primers used for qRT-PCR of *ZmDCLs* and *ZmAGO2* mRNAs.
(XLS)

Table S7 The probes used for Northern blotting of vsiRNAs.
(XLS)
